# Twenty-five year experience with aortic valve-sparing root replacement in a single teaching center

**DOI:** 10.3389/fcvm.2023.1104149

**Published:** 2023-03-10

**Authors:** Juri Sromicki, Mathias Van Hemelrijck, Martin O. Schmiady, Matthias Greutmann, Francesca Bonassin Tempesta, Carlos-A. Mestres, Paul R. Vogt, Thierry P. Carrel, Tomáš Holubec

**Affiliations:** ^1^ Department of Cardiac Surgery, University Zurich and University Hospital Zurich, Zurich, Switzerland; ^2^ Department of Cardiology, University Zurich and University Hospital Zurich, Zurich, Switzerland; ^3^ Department of Cardiovascular Surgery, Goethe University Frankfurt and University Hospital Frankfurt, Frankfurt, Germany

**Keywords:** aortic valve sparing surgery, aortic root reimplantation, aortic regurgitation (AR), aortic aneurysm (AA), David operation and David procedure, david operation, david procedure

## Abstract

**Objectives:**

Aortic valve-sparing root replacement (AVSRR) is a technically demanding procedure. In experienced centers it offers excellent short- and long-term results, making the procedure an attractive alternative for aortic root replacement especially in young patients. The aim of this study was to analyze long-term results of AVSRR using the David operation in our institution over the last 25 years.

**Methods:**

This is a single-center retrospective analysis of outcomes of David operations performed in a teaching institution not running a large AVSRR-program. Pre-, intra- and postoperative data were collected from the institutional electronic medical record system. Follow-up data were collected through direct contact of the patients and their cardiologists/primary care physicians.

**Results:**

Between 02/1996 and 11/2019, 131 patients underwent David operation in our institution by a total of 17 different surgeons. Median age was 48 (33–59), 18% were female. Elective surgery was performed in 89% of the cases, 11% were operated as emergency in the setting of an acute aortic dissection. Connective tissue disease was present in 24% and 26% had a bicuspid aortic valve. At hospital admission 61% had aortic regurgitation grade ≥3, 12% were in functional NYHA-class ≥III. 30-day mortality was 2%, 97% of the patients were discharged with aortic regurgitation ≤2. In 10-year follow-up, 15 (12%) patients had to be re-operated because of root-related complications. Seven patients (47%) received a transcatheter aortic valve implantation, 8 (53%) required surgical replacement of the aortic valve or a Bentall-De Bono operation. Estimated reoperation-free survival at 5 and 10 years was 93.5% ± 2.4% and 87.0% ± 3.5%, respectively. Subgroup analysis showed no differences in reoperation-free survival for patients presenting with a bicuspid valve or preoperative aortic regurgitation ≥3. However a preoperative left ventricular end diastolic diameter of ≥5.5 cm was associated with worse outcome.

**Conclusion:**

David operations can be performed with excellent perioperative and 10-year follow-up outcomes in centers not running large AVSRR-programs.

## Introduction

In patients with aortic root aneurysm and aortic valve (AV) dysfunction root- and AV-replacement with a composite valved graft, as introduced by Bentall and De Bono in 1968, has proven to be a safe and durable procedure (1). However, younger patients with aortic root-dilatation and a structurally normal AV may benefit from an aortic valve-sparing root replacement (AVSRR), since valve replacement in these cases is inevitably associated with life-long oral anticoagulation, an increased risk of infective endocarditis and redo-surgery in case a tissue valve has been selected.

To preserve the AV in case of a dilated sinus of Valsalva, several surgical techniques have been introduced during the last three decades (2). In the remodeling-technique, such as the Yacoub-procedure, the vascular prosthesis to replace the aortic root is trimmed to provide three artificial sinuses, while the annulus is not stabilized as well as in the reimplantation technique. In the latter, the native aortic valve is resuspended into the vascular prosthesis and the aortic annulus is stabilized through sub-annular sutures. The Yacoub-procedure seems to offer very nice functional results with a preserved elasticity of the aortic root and of the native aortic annulus. This advantage may be outweighed through a late enlargement of the aortic annulus leading to progressive aortic regurgitation, particularly in patients with underlying connective tissue disease, for whom the Yacoub-technique is not recommended. The reimplantation technique, originally described by David et al. in 1992 (3, Central image) allows less expansion of the annulus during the cardiac cycle (4), but offers excellent short- and long-term-results when performed in experienced centers (5).

**CENTRAL IMAGE:**
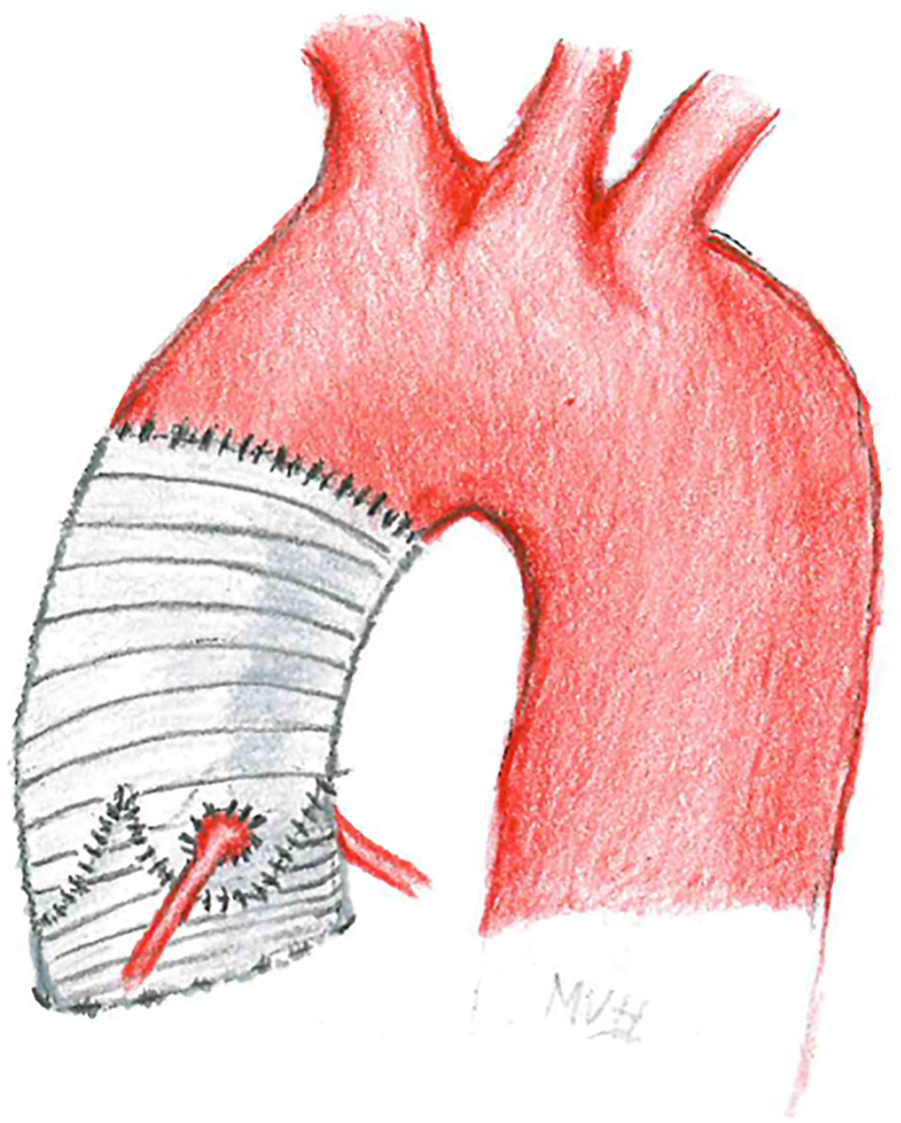
Aortic valve sparing root replacement – David operation.

Short- and long-term analyses for both mentioned AVSRR-procedures have mostly been reported from high-volume centers running larger AVSRR programs in which the procedures are performed by few specialized surgeons. Little is known if these technically demanding procedures can be performed with similar results in a mid-volume teaching center. The aim of this study was to analyze the long-term-results of AVSRR using the David operation in our institution over the last 25 years.

## Methods

### Study design

This is a retrospective analysis from a single-center's database reporting the results of patients that were treated with an aortic valve sparing root replacement as originally described by David et al. in 1992 (3).

### Ethics

The study design was approved by the institutional review board/ethics committee (KEK-ZH # 2015-0292). Informed consent was waived due to the retrospective nature of the study and institutional database approval.

### Operative technique

All patients underwent AV reimplantation into a tubular graft (3). Minor modifications of the procedure, e.g., plication of the free edge of the leaflets at the presence of valve-prolapse, were left at the discretion of the operating team (6). Additional procedures were performed whenever indicated and included coronary bypass-surgery, additional valve-surgery or (partial) replacement of the aortic arch. Despite technically possible through minimally invasive access (7), for the sake of optimal exposure and considering the potential need for concomitant procedures, all David operations in this study were performed through a full sternotomy.

### Indication for surgery

Indication for aortic root replacement was based on elective echocardiographic assessment for chronic findings or on intraoperative transesophageal echocardiography in case of an emergency-situation such as acute aortic dissection. Indication for AVSRR was made intraoperatively in these cases. Generally, patients with normal valve opening, absence of significant valve-calcifications and aortic root enlargement were identified as potential candidates for a David operation.

### Inclusion/exclusion criteria

Attempted David operations with intraoperative conversion to a Bentall—De Bono operation were not included in this analysis.

### Definitions

Indication for surgery was based on the ESC/EACTS guidelines valid on the respective date of admission (8). Valve regurgitation was assessed by transthoracic or transesophageal echocardiography as recommended in the ESC guidelines on the management of valvular heart disease. The anatomy of the AV (bicuspid or tricuspid) was assessed preoperatively, whenever possible, but had to be confirmed intraoperatively in order to be classified as tri- or bicuspid. A partial fusion of two leaflets was classified as a bicuspid valve when described so in the operating report.

Samples of the aortic wall were sent for histopathological analysis and in certain cases for genetic testing for connective tissue-disease (CTD). Patients were classified to have a CTD if diagnosis was highly suggestive due to concomitant disease or presenting phenotype, hereditary predisposition or if CTD was confirmed by genetic testing. Acute type A aortic dissection was defined as in the mentioned guidelines above (8).

Re-operation during follow-up was defined as surgery related to the aortic valve or aortic root and included transcatheter aortic valve implantation (TAVI) as well as conventional surgical procedures. Interventions beyond the aortic arch, e.g., thoracic endovascular aortic repair (TEVAR) in case of residual dissection or dilatation of the descending thoracic aorta, were not considered as reoperations. Early death was defined as 30-day or in-hospital death with interhospital transfer not considered as hospital discharge.

### Data collection

All patients had pre-operative assessment by transthoracic and/or transesophageal echocardiography as well as by computed tomography or magnetic resonance imaging. Severity of pre-operative aortic valve regurgitation was abstracted from echocardiography reports and in case of ambiguity, echocardiographic studies were reviewed for the purpose of this study. Pre-, intra- and postoperative data were collected from the institutional electronic medical record system. Clinical follow-up data were collected from outpatient clinic visits or by directly contacting the patients and their primary care physicians, echocardiographic follow-up data were mainly derived from transthoracic echocardiography performed by in-house or patients’ private cardiologists. The database was locked as of December 2019 for completion of follow-up.

### Outcomes of interest

Main goal of this study was to describe reoperation free long-term survival after AVSRR in our center. Short- and long-term performance of the reimplanted aortic valve was defined as secondary outcome of interest. Potential risk-factors for adverse outcomes (in-hospital mortality, long-term-mortality, reoperation) were investigated in subgroup analyses.

### Statistical analyses

Standard descriptive statistics were used to summarize data. Continuous and discrete variables are presented as means with standard deviation or median and 25%/75% Quartile when not normally distributed. Categorical and ordinal variables are presented as absolute numbers and proportions.

Survival and freedom from events were calculated according to the Kaplan–Meier method. The log-rank test with Kaplan–Meier curves was used for group-survival-comparisons. The estimated survival of a patient started at the time of the operation and ended at the time of death/reoperation (event) or the latest known follow-up (censored). Cox-regression models were used for risk factor analysis to confirm significant log-rank tests.

A two-sided *p*-value <0.05 was considered statistically significant. Statistical analyses were performed using the SPSS 25.0 software package (SPSS, Inc., IL, United States).

## Results

### Preoperative data

Between February 1996 and November 2019, 131 patients (18% female) underwent AVSRR using the David technique by a total of 17 different surgeons [Median of surgeries performed per surgeon: 4 (1–13)]. Median age at time of surgery was 48 years (38–59). In a total of 31 (24%) patients CTD was identified as the underlying cause of aortic root dilatation. Three different CTD were observed in this study: Marfan's disease, Loeys-Dietz and Ehlers-Danlos syndromes. The majority of operations were elective procedures (89%) but emergency operation for acute type A dissection (ATAAD) comprised 11% of cases. In 13 patients (10%), AVSRR was performed as a cardio-thoracic reoperation: five patients had prior surgery for coarctation of the aorta, one patient had surgical repair for an acute type B aortic dissection, three patients suffered progressive root dilatation after supracoronary replacement of the ascending aorta, one patient had progressive regurgitation after Yacoub-remodeling, three patients had severe regurgitation of the neo-aortic valve after Ross-procedure. A bicuspid valve (Sievers type 0/1/2) (9) was diagnosed in 26% of the patients. Eighty-eight percent of the patients were asymptomatic or had only minor symptoms (NYHA-functional class I & II) prior to hospital admission, moderate or severe aortic regurgitation was present in 61% of the patients. The mean diameter of the sinus of Valsalva was 50 ± 7 mm, the mean diameter of the ascending aorta at the level of the pulmonary bifurcation was 47 ± 13 mm respectively. Data on baseline characteristics, comorbidities and measurements from preoperative echocardiography/computed tomography are presented in Table 1.

**Table 1:** Preoperative characteristics.

**Age (years)**	48 (33–59)
**Gender**	
Male	107 (82%)
Female	24 (18%)
**Comorbidities**	
Connective tissue disease	30 (23%)
Insulin dependent DM	2 (2%)
Arterial Hypertension	53 (40%)
Peripheral artery disease	4 (3%)
COPD	3 (2%)
Cerebrovascular event	7 (5%)
Acute Type A aortic dissection	15 (11%)
Redo heart-surgery	13 (10%)
**Laboratory findings**	
Preoperative Creatinine (µmol/L)	82 ± 16
**NYHA functional class**	
NYHA I	91 (72%)
NYHA II	20 (16%)
NYHA III	11 (9%)
NYHA IV	4 (3%)
**Echo- and CT-data**	
Cuspidity	
Bicuspid valve	34 (26%)
Tricuspid valve	97 (74%)
Aortic regurgitation	
None (0)	12 (9%)
Trivial (1)	15 (12%)
Mild (2)	23 (18%)
Moderate (3)	50 (39%)
Severe (4)	29 (22%)
LVEF (%)	60 ± 8
LV-Diameter	
LVESD (mm)	36 ± 8
LVEDD (mm)	56 ± 9
Aortic diameters	
Annulus (mm)	27 ± 5
Aortic root (mm)	50 ± 7
ST-junction (mm)	44 ± 9
Ascending aorta (mm)	47 ± 13

### Intraoperative data

All 131 patients underwent successful aortic valve reimplantation. Mean cardiopulmonary bypass and arrest times were 186 ± 73 and 135 ± 48 min respectively. A central suture-plication at the *nodulus Arantii* was performed in 39 patients (30%). A Dacron-graft from 22 to 32 mm in diameter was used for AVSRR. Sixteen patients (12%) required an open distal anastomosis during a short period of circulatory arrest with antegrade cerebral perfusion. Additional valve repair/replacement (3 mitral repair, 1 mitral replacement, 1 pulmonary homograft) was needed in 5 patients (4%), concomitant aortocoronary bypass was performed in 9 patients (7%).

### Early postoperative outcomes

Three patients (2%) died in the early postoperative period. One patient suffered from hypoxic brain damage in the setting of an ATAAD, one patient died due to an acute bleeding from the aortic root resulting in cardiac tamponade and prolonged resuscitation and one patient died due to multi-organ-failure in the setting of ATAAD with consecutive open-chest-treatment because of hemodynamic instability and bleeding. Four patients (3%) suffered from perioperative stroke, 5 patients (4%) required the implantation of a permanent pacemaker. Ninety-seven percent of the patients were discharged from hospital with aortic regurgitation ≤II. The mean AV-gradient at discharge was 8 ± 4 mmHg. Intraoperative and discharge data are presented in Tables 2, 3.

**Table 2:** Intraoperative data.

**CPB-time (min)**	186 ± 73
**Aortic cross-clamp time (min)**	135 ± 48
**Graftsize (mm)**	28 ± 2
**Plications performed**	
Overall	39 (30%)
Noncoronary cusp	27 (21%)
Right coronary cusp	25 (19%)
Left coronary cusp	25 (19%)
**Additional procedures**	
+ ACBP	9 (7%)
+ Other Valve	5 (4%)
+ Hemiarch	11 (8%)
+ Arch	5 (4%)

**Table 3:** Discharge data.

**In-hospital mortality**	3 (2%)
**Perioperative cerebrovascular event**	4 (3%)
**Perioperative pacemaker-implantation**	5 (4%)
**Echocardiography at discharge**	
Aortic regurgitation	
None (0)	42 (33%)
Trivial (1)	59 (46%)
Mild (2)	22 (17%)
Moderate (3)	4 (3%)
Severe (4)	0 (0%)
LVEF (%)	57 ± 8
LV-Diameter	
LVESD (mm)	35 ± 8
LVEDD (mm)	52 ± 7
Aortic valve gradient	
dPmax (mmHg)	16 ± 8
dPmean (mmHg)	8 ± 4
Aortic diameters	
Annulus (mm)	23 ± 2
Aortic root (mm)	33 ± 4
Ascending aorta (mm)	31 ± 4

### Long-term outcomes

The completeness of follow-up was 99%. Only one patient was lost to follow-up due to moving abroad. Median follow-up-time was 8.7 years (6.2–12.9). Eighteen patients (14%) died during the follow-up, 15 patients (12%) had to be re-operated due to valve or graft-related complications at a median of 10.7 years (6.1–15.6) after initial David operation. Reasons for reoperation were: aortic regurgitation (6 patients, 40%), aortic stenosis (7 patients, 47%) and aortic valve endocarditis (2 patients, 13%). Of the 15 patients that needed reoperation/reintervention, 7 (47%) received a TAVI, 8 (53%) patients underwent redo-surgery including aortic valve replacement. Median age at reintervention/reoperation was 73.8 years (64.2–79.4) and 46.6 years (27.7–60.3) respectively. There were no cases of perioperative mortality for both approaches.

**Figure 1:**
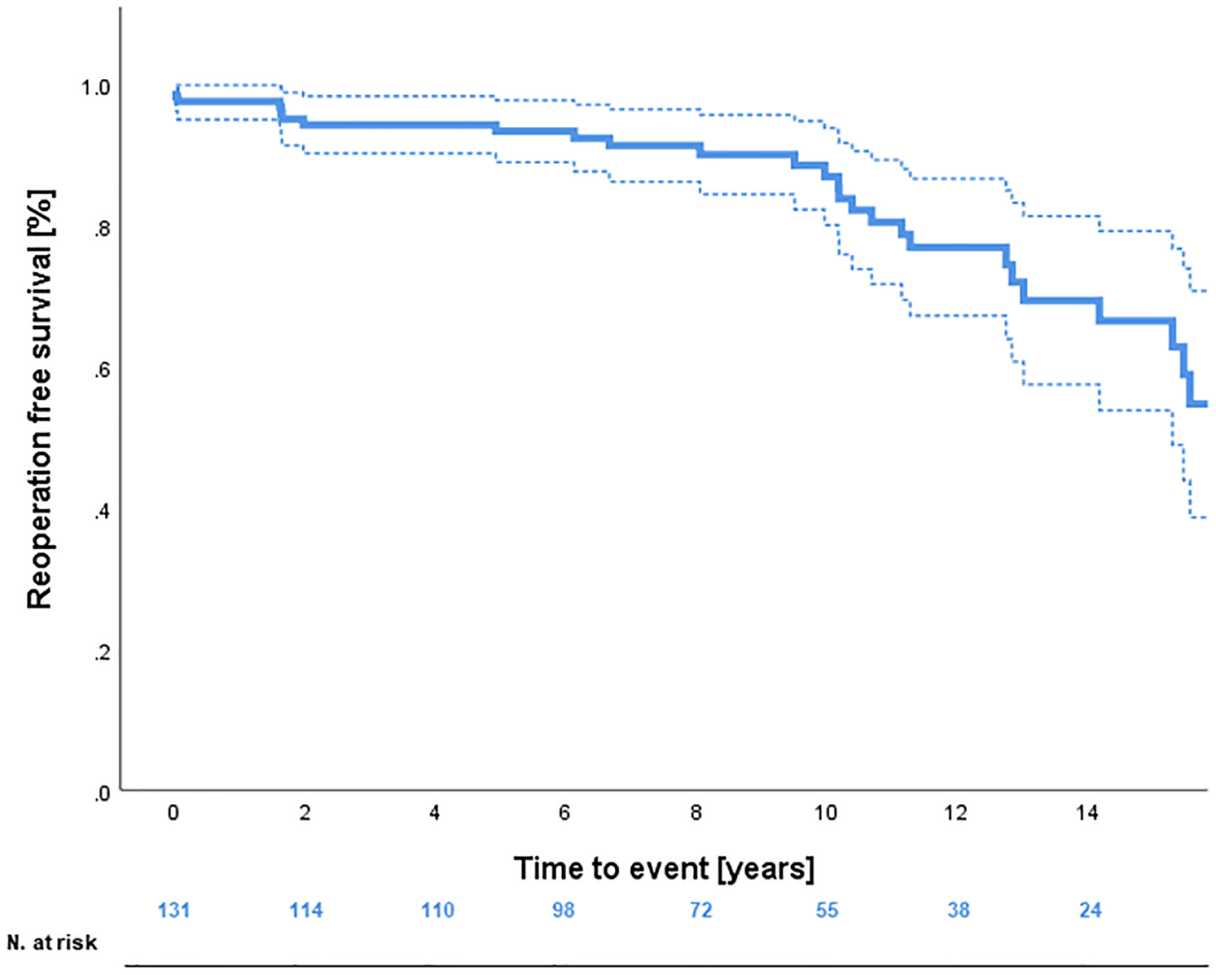
Reoperation free survival after AVSRR with 95% confidence-interval.

**Figure 2:**
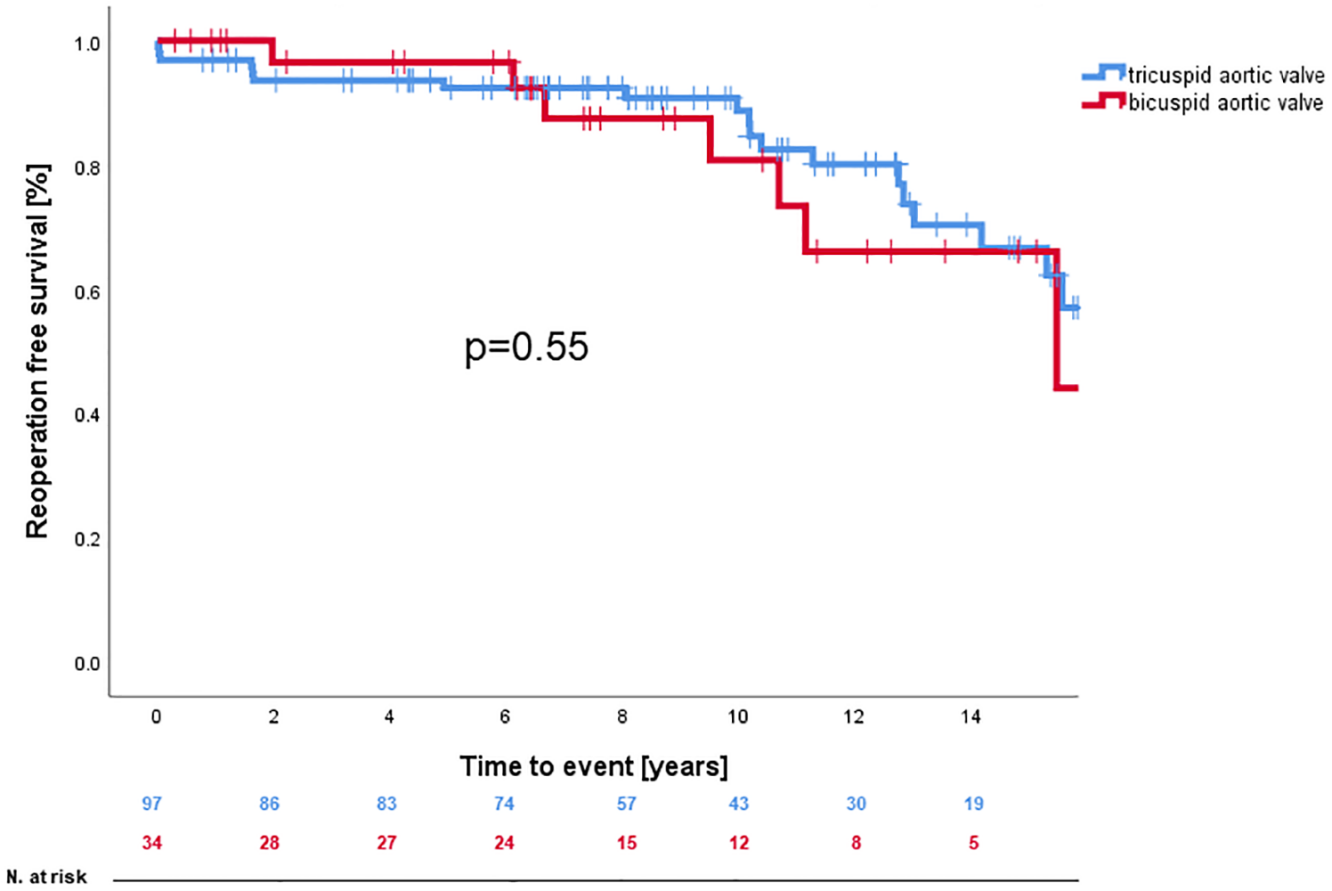
Reoperation free survival after AVSRR with bicuspid vs. tricuspid valve.

**Figure 3:**
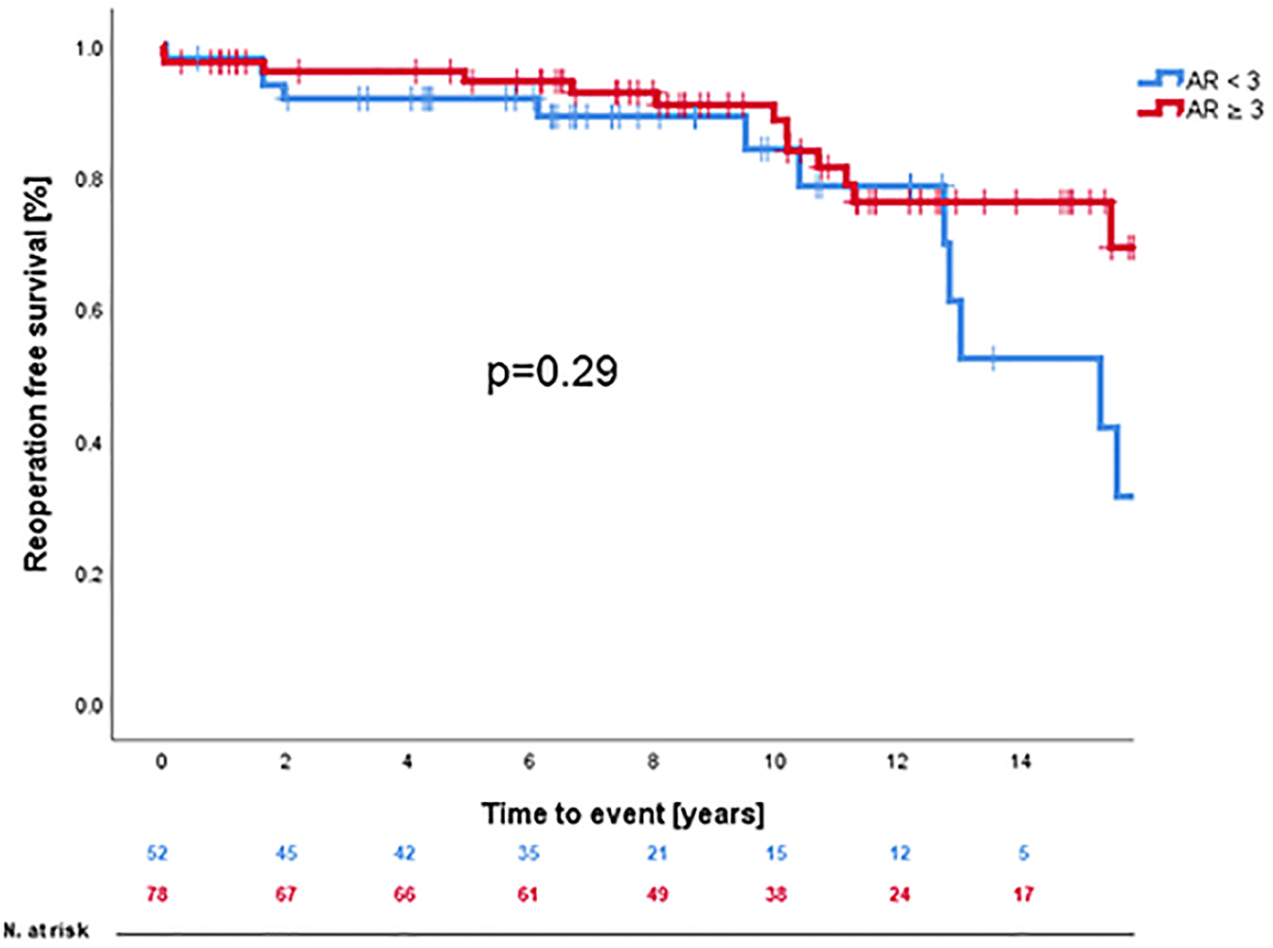
Reoperation free survival after AVSRR with aortic regurgitation ≥3 vs. <3.

**Figure 4:**
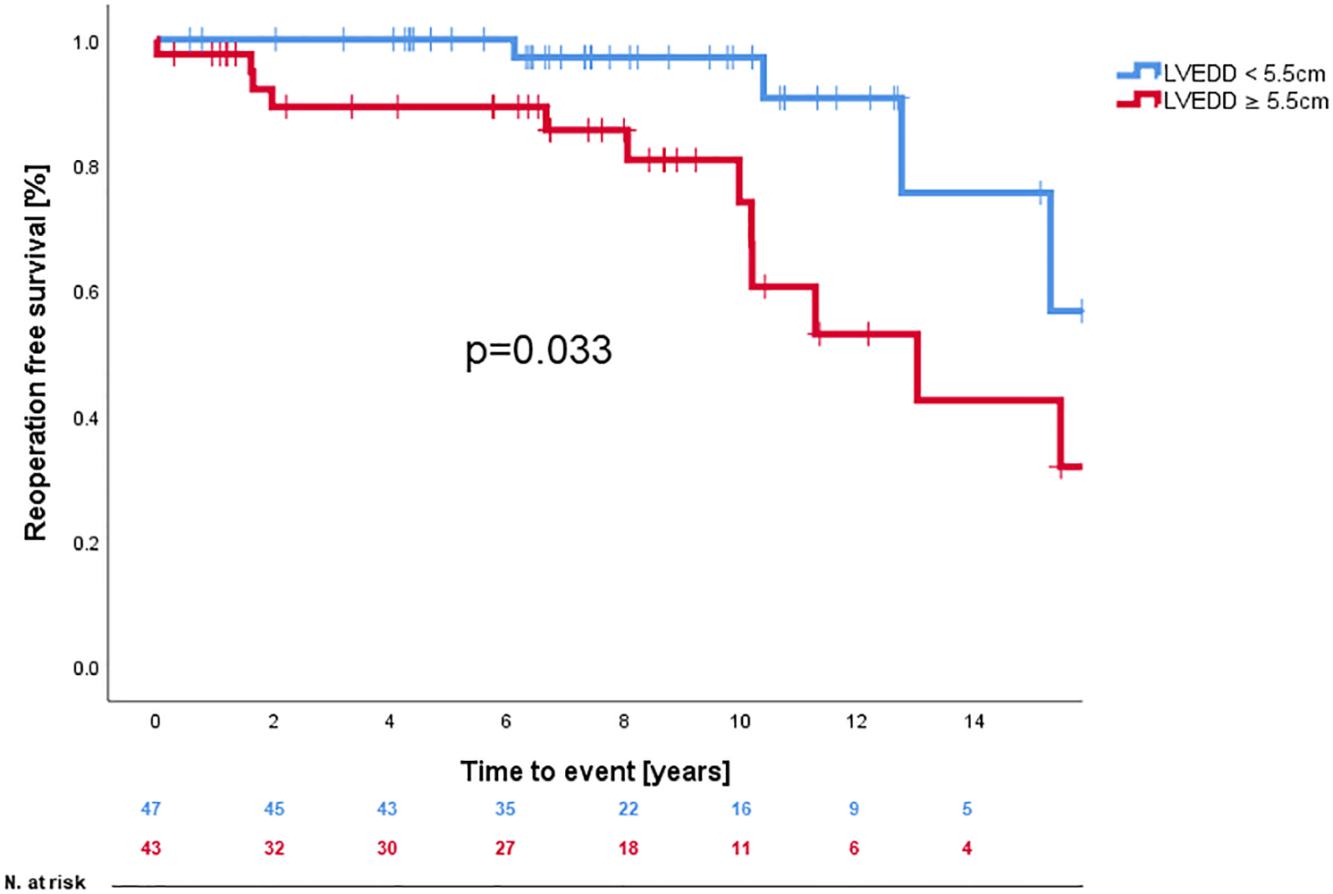
Reoperation free survival after AVSRR with LVEDD <5.5 vs. ≥5.5 cm.

A Kaplan-Meier analysis showing reoperation-free survival-rates is provided in Figure 1. Reoperation-free survival at 1, 5, 10, and 15 years was 97.7% ± 1.3%, 93.5% ± 2.4%, 87.0% ± 3.5%, and 66.6% ± 6.5%, respectively. Log-rank-test revealed no difference in reoperation free survival between AVSRR in bicuspid vs. tricuspid valve (*p* = 0.55, Figure 2) or AVSRR in case of preoperative aortic regurgitation ≥3 vs. <3 (*p* = 0.29, Figure 3). However, reoperation free survival was significantly longer in patients without left ventricular dilatation [left ventricular end diastolic diameter (LVEDD) <55 mm vs. LVEDD ≥ 55 mm] at the time of AVSRR [log-rank-test *p* = 0.033/Cox-regression model *p* = 0.027, HR 3.583 (1.152–11.143), Figure 4].

**Figure 5:**
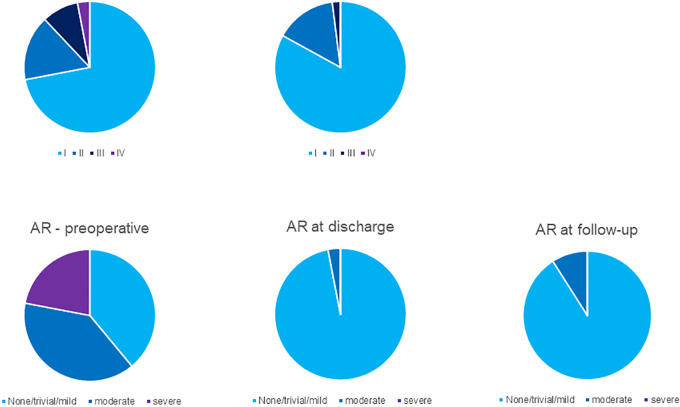
NYHA functional class and aortic regurgitation preoperative, postoperative and in late follow-up.

At latest follow-up, 100 patients were alive and did not require a re-operation. Clinical and echocardiography-data were obtained from all of them (100%). Median follow-up time for this patient-group was 8.2 years (6.1–12.2) for the clinical assessment and 7.2 years (4.3–10.7) for the echocardiography-data. In follow-up, 98% of the patients were in functional NYHA Class I or II. Eight years after surgery 91% of the patients remained with aortic regurgitation ≤2 (Figure 5). Left ventricular ejection fraction and diameters at the level of the aortic annulus, the sinus of Valsalva, the ascending aorta as well as left ventricular end diastolic diameter remained stable over the years. Clinical and echocardiographic follow-up data are summarized in Table 4.

**Table 4:** Follow-up data.

**Clinical follow-up (*n* = 130)**	
Duration clinical follow-up (years)	8.7 (6.2–12.9)
Status at follow-up	
Alive	100 (76%)
Dead	18 (14%)
Reoperated	15 (12%)
Reoperated and dead	2 (1%)
Postoperative cerebrovascular event	4 (4%)
**Clinical follow-up—alive and not reoperated (*n* = 100)**	
Duration clinical follow-up (years)	
NYHA Functional class	8.2 (6.1–12.2)
NYHA I	83 (83%)
NYHA II	15 (15%)
NYHA III	2 (2%)
NYHA IV	0 (0%)
**TTE follow-up—alive and not reoperated (*n* = 100)**
Duration TTE follow-up (years)	7.2 (4.3–10.7)
Aortic regurgitation	
None (0)	19 (19%)
Trivial (1)	35 (35%)
Mild (2)	37 (37%)
Moderate (3)	9 (9%)
Severe (4)	0 (0%)
LVEF (%)	61 ± 6
LV-Diameter	
LVESD (mm)	33 ± 7
LVEDD (mm)	51 ± 7
Aortic valve gradient	
dPmax (mmHg)	13 ± 10
dPmean (mmHg)	8 ± 7
Aortic diameters	
Annulus (mm)	24 ± 3
Aortic root (mm)	34 ± 4
Ascending aorta (mm)	32 ± 4

## Discussion

Aortic valve sparing root replacement is considered the optimal treatment option for significant aortic root dilatation in case of suitable valve anatomy, especially in young patients. When performed in experienced centers with a well-structured AVSRR program, excellent results can be expected in adolescents as well as younger adults with reoperation-free survival rates above 70% in 5 to 15-year follow-up (10–13). In this study, we demonstrated, that comparable results can be obtained in a teaching-center as well, where AVSRR-surgeries are performed by way more different surgeons than in specialized centers. The results achieved in our institution do not diverge much from results in clinics with highest expertise in AVSRR.

AVSRR using the David technique is a demanding operation, usually performed by experienced surgeons. As in every other, technically demanding procedure, it is known that the surgeon's experience has a direct impact on early- and long-term outcomes of AVSRR procedures (14). Still it is noteworthy, that not only the surgeons experience is crucial to achieve best possible results. Critical patient selection with precise preoperative assessment of the aortic root as well as close aftercare of the patients, especially in case of a dilated left ventricle (15), is mandatory for procedural and long-term success.

Twenty four percent of the patients in this study had an underlying CTD, a substantial proportion (26%) presented with a bicuspid valve and 21% of the cases were performed under aggravating circumstances such as reoperative heart surgery or in the setting of an ATAAD. Although good long-term-results can be achieved with AVSRR in emergency surgery for aortic root-related diseases, AVSRR poses an increased risk for additional complications and poor long-term-outcomes when performed in this condition (16). This is especially relevant in case of unsatisfactory root reconstruction when the aortic root eventually has to be replaced by a composite valve graft in a second cardiopulmonary-bypass run. This study does not include an intention-to-treat analysis and therefore the question, if AVSRR should be considered in these kinds of extraordinary settings cannot be answered.

In our study, reoperation-free long-term survival was significantly lower once left ventricular end diastolic diameter exceeded 55 mm. This indicates, that decision on operative timing in non-ATAAD should not only be based on symptoms or degree of aortic regurgitation, but also on left ventricular dilatation as it is stated in the current european guidelines for the management of valvular heart disease (17).

The main expected advantages of a valve sparing procedure are the lower risk of infective endocarditis compared to prosthetic valve replacement and no need for long-term anticoagulation as required after implantation of mechanical prostheses. This finding made AVSRR surgery an attractive treatment option for younger patients, especially when it can be expected that the re-implanted valve may last longer than a biological prosthesis. However, 15% of the patients treated with the David operation had to be re-operated during follow-up, mostly due to valve related complications thus underscoring the need for careful patient selection and surveillance during follow-up. Relapse of significant aortic regurgitation was observed almost as frequent as the development of aortic stenosis. Reoperation for valve-related problems is feasible, however associated with an increased surgical risk due to mediastinal scaring and adhesions. For this reason, in this study, almost half of the patients that needed reoperation/reintervention due to valvular problems, were selected for transcatheter valve implantation (18).

Despite technical challenges especially in asymmetrical commissural orientation (19), both bicuspid as well as tricuspid valves can be re-implanted with very good results. A precise assessment of the aortic root geometry is crucial to successful treatment (20). Minor corrections on the free edge of the leaflets can be performed using central suture plications at the *nodulus Arantii*, a technique overall used in 30% of the patients undergoing AVSRR in this study (32% in tricuspid valves, 24% in bicuspid valves). No differences in reoperation-free long-term survival were observed comparing patients that were treated with leaflet-plications to those without correction of leaflet-prolapse.

### Strengths and limitations

The strength of this study is in the 99% completeness of follow-up at our clinic or referring physicians. Knowing that the possible onset of aortic valve deterioration or significant AV regurgitation need close monitoring, patients after AVSRR are checked for AV-dysfunction or left ventricular dilatation on a yearly basis.

This study has some limitations, foremost the retrospective study design and the long study period in which perioperative care, perfusion techniques etc. might have changed over time. Group-heterogeneity may confound definitive conclusions. Despite data collection for this study was carefully done by one person applying the exact criteria outlined in the methods-section, echocardiography-data were assessed by different cardiologists following different protocols and may therefore contribute to selection bias. The operations analyzed in this study were performed by a total of 17 different surgeons over a long period of time. Individual experience as well as individual treatment concepts may also have a direct impact on patient-outcomes. However, the low inclusion rate and heterogeneity in the indications, procedures and results of this study may actually represent the outcomes of AVSRR in low volume centers and directly support the feasibility of the procedure even if it's actually performed under training-conditions by different surgeons.

Additionally it needs to be underlined, that only a limited number of patients reached long-term follow-up >10 years. Especially in the context of alternative replacement of the aortic valve with a bio-prosthesis, which nowadays is expected to last longer than 10 years, conclusions weather AVSRR is superior in long-term follow-up need to be critically evaluated.

## Conclusion

Despite being a technically demanding procedure, AVSRR using the David operation can be performed safely in mid-volume centers with excellent perioperative and 10-year follow-up outcomes. The results presented in this study may justify the use of AVSRR-surgery in well evaluated younger patients in centers where only a handful of cases are performed yearly. However, being a time-consuming procedure, dependent on preoperative planning, David operations should preferably be perfomed in elective settings or remain in experienced hands, under aggravating circumstances such as redo-heart-surgery or ATAAD.

In 10 year-follow-up we experienced few root related complications and the majority of the patients remained asymptomatic with stable root diameters and non-significant aortic regurgitation over time. Aortic valve sparing procedures offer a safe alternative for complete root replacement especially in younger patients in whom the intake of oral anticoagulation is undesirable.

## Data Availability

The original contributions presented in the study are included in the article/Supplementary Material, further inquiries can be directed to the corresponding author.
